# Salt Reduction and Iodine Fortification Policies Are Compatible: Perspectives for Public Health Advocacy

**DOI:** 10.3390/nu16152517

**Published:** 2024-08-01

**Authors:** Jessica Rigutto-Farebrother, Michael B. Zimmermann

**Affiliations:** 1 Human Nutrition Laboratory, Institute of Food, Nutrition and Health, ETH Zürich, 8092 Zürich, Switzerland; 2 Laboratory of Nutrition and Metabolic Epigenetics, Institute of Food, Nutrition and Health, ETH Zürich, 8092 Zürich, Switzerland; 3 MRC Human Immunology Unit, MRC Wetherall Institute of Molecular Medicine, University of Oxford, John Radcliffe Hospital, Oxford OX3 9DS, UK

**Keywords:** fortification, iodine, salt, sodium, public health

## Abstract

Cardiovascular diseases account for almost 18 million deaths annually, the most of all non-communicable diseases. The reduction of dietary salt consumption is a modifiable risk factor. The WHO recommends a daily sodium intake of <2000 mg but average consumption exceeds this in many countries globally. Strategies proposed to aid effective salt reduction policy include product reformulation, front of pack labelling, behavioural change campaigns and establishing a low-sodium-supportive environment. Yet, salt for household and processed food use is, in countries wholly or partially adopting a universal salt iodisation policy, the principal vehicle for population-wide iodine fortification. With salt reduction policies in place, there is concern that iodine deficiency disorders may re-emerge. Recognising the urgency to tackle the rising prevalence of NCDs yet not risk the re-emergence and detrimental effect of inadequate iodine intakes, this review lays out the feasibility of integrating both salt reduction and salt iodine fortification strategies. Reducing the burden of health risks associated with an excessive sodium intake or inadequate iodine through population-tailored, cost-effective strategies involving salt is both feasible and achievable, and represents an opportunity to improve outcomes in public health.

## 1. Introduction

The World Health Organisation promotes both the implementation of programmes to reduce population sodium intake as one of the cost-effective strategies to lessen the burden of non-communicable diseases (NCD) and universal salt iodisation to prevent and control iodine deficiency disorders. However, while salt reduction is recommended globally, there is concern that iodine deficiency disorders may re-emerge. However, through a targeted and combined approach, salt reduction and iodine fortification together represent an exceptional opportunity. Reducing the burden of health risks associated with an excessive sodium intake or inadequate iodine through population-tailored, cost-effective strategies involving salt is both achievable and easy to implement and represents a considerable double duty opportunity to improve outcomes in public health.

The WHO has defined double-duty actions as those interventions, programmes, and policies that have the potential to simultaneously reduce the risk or burden of both undernutrition (i.e., iodine deficiency) and diet-related NCDs (such as hypertension). Double-duty actions are not necessarily new actions; they are often actions that are already used to address single forms of malnutrition but with the potential to address multiple forms simultaneously in an integrated solution [1].

Recognising the urgency to tackle the rising prevalence of NCDs yet not risk the re-emergence and detrimental effect of inadequate iodine intakes, this review aims to lay out the feasibility of integrating both salt reduction and salt iodine fortification strategies.

## 2. Salt Reduction

NCDs are responsible for 71% of all deaths worldwide, with almost three quarters occurring in low- and middle-income countries [2]. The main drivers of NCDs are four modifiable risk factors [2]: the harmful use of alcohol, tobacco use, physical inactivity, and unhealthy diets dominated by foods high in sugar, saturated and trans-fats, and excessive salt consumption—which makes NCDs largely preventable. In 2013, the World Health Organisation (WHO) launched the global action plan for the prevention and control of NCDs 2013–2020 to take coordinated action at all levels across nine voluntary global targets that include a 30% reduction in salt consumption by 2025 [3]. Salt is particularly important since it is one of the major modifiable risk factors for cardiovascular diseases including hypertension, heart attack, and stroke that account for almost 18 million deaths annually, the most from NCDs [2]. Directly, 4.1 million annual deaths globally have been attributed to excess salt intake [4].

The morbidity associated with high blood pressure alone is expensive and estimated to cost billions. Patients in the US pay an additional US$2000 annually in healthcare costs, totalling US$131 billion [5], and in a review of 15 lower-middle, upper-middle- and high-income countries (using the World Bank Classification) the mean total costs per person were I$630, ranging from I$1190 (Jamaica) to I$70 (Indonesia) [5]. However, in in low- and middle-income countries (LMICs), the true burden of high blood pressure on public health and individual costs is unclear [6].

The WHO recommends a daily sodium intake for adults of less than 2000 mg per day [equivalent to 5 g, or a teaspoon, of salt] [7,8], adjusted downwards for children based on energy requirements [8]. Average salt intakes in many countries are still well above this, though several strategies have been shown to be cost effective (≤I$100 per Disability-Adjusted Life Year (DALY) averted in LMICs) for salt intake reduction [9] including the following:

- Food product reformulation to contain less salt and target setting for the amount of salt in foods and meals;
- The establishment of a supportive environment for the provision of low-sodium options in public institutions, e.g., hospitals, schools, workplaces, and nursing homes;
- Behavioural change communication and mass media campaigns;
- Implementation of front-of-pack labelling.

### 2.1. Food Product Reformulation to Contain Less Salt

Food product reformulation policies to reduce sodium content have been adopted in 65 WHO member states, of which 27 have adopted some level of mandatory policy [8]. Following mass media campaigns, it is the most popular strategy to date. Reformulation involves the changing of the composition of a processed food to achieve a food with a lower sodium content. It is distinct from food enrichment or food fortification and considered a key option in the fight to lower population salt intakes [10]. Product reformulation and target setting to limit salt in foods and meals is a successful strategy with a high cost-effectiveness; modelling suggests incremental cost-effectiveness ratio cost reductions of up to I$17,243 per Quality-Adjusted Life Year (QALY), I$62,896 per life year gained, and I$9798 per DALY for voluntary or mandatory reformulation [11]. Modelling from the United Kingdom suggests a £1.44 10-year return on investment for every £1 spent [12], and cost savings of >£660 million with mandatory product reformulation [13]. Furthermore, product reformulation shows potential for impact across all socioeconomic levels of society [14].

Product reformulation may include the removal of sodium from processed food products, and in categories including bread and baked goods, breakfast cereals, processed meats, cheese, chips, soups, and sauces, reductions of up to 40% have been successful [10]. It may be a particularly successful strategy in high-income countries, where over 70% of dietary salt is consumed in processed food [15,16], but also in lower-income countries. As countries proceed through economic and nutritional transition, processed foods such as bread, meat products, instant noodles, preserved foods, and condiments also become commonly consumed sources of dietary salt that can be subject to product reformulation [17].

Product reformulation also includes the use of substitutes that may help to reduce sodium content without a loss in flavour. Substitution with potassium chloride is a potential route for product reformulation that could be suitable for adoption in both higher-and lower-income countries [15,16,17]. Use of potassium chloride as a salt replacer has been shown to lessen increases in blood pressure over time [18], and modelling analyses also suggest that it is cost-effective, with the forecasted healthcare savings rising to a potential US$14,545,300 per annum [19]. Potassium chloride as a salt substitute could not only lower sodium but also increase potassium consumption towards the WHO recommendation of 3510 mg/day in adults [20,21]. Increasing evidence supports the protective role of potassium against hypertension and cardiovascular disease [22].

In China, a low-sodium substitute that reduced sodium chloride in household salt by 35% led to a greater decrease in blood pressure across a three-year study period compared to normal salt [18]. In Taiwan, a potassium-enriched salt substitute (49% KCl, 49% NaCl, and 2% other additives) led to a 41% lower risk of cardiovascular disease after 31 months compared to normal salt [23]. In Vietnam, where salt, fish sauce, and bot canh contribute to about 70% of dietary sodium intakes, modelling of partial replacement of sodium chloride (salt) with potassium chloride in these products delivered yearly cost savings of US$205,764 for the voluntary strategy and US$14,545,300 for a government-regulated strategy and effectiveness benefits of 0.009 incremental QALYs gained for the voluntary strategy and 0.074 QALYs gained for the regulated strategy [19]. In this analysis, the annual cost of implementing these strategies was ≤US$0.02 per capita but led to a reduction in stroke incidence of between 32,595 and 2,366,480 and cardiovascular events of between 22,830 and 1,648,590 over the 70-year model lifetime [19].

Potassium chloride has a Generally Recognized As Safe (GRAS) status as a multipurpose ingredient in foods [24], and its use in substitution is considered safe concerning with respect to dietary potassium intakes in the general population [21]. Caution may be needed for those with impaired potassium excretion such as patients with chronic kidney disease [25]. Furthermore, the use of potassium chloride as a salt substitute may hinder population taste adaptation to lower salt thresholds and therefore slow the progress of salt reduction policies, and therefore should only be considered where a reduction of saltiness could be detrimental to the physical or organoleptic properties of foods. Though potassium chloride is already successfully used in foods as a flavour enhancer or salt substitute, the possibility of its fortification with iodine is, to date, an open research priority [26].

### 2.2. Establishment of a Supportive Environment

In public institutions such as hospitals, schools, workplaces, and nursing homes, for the provision of low-sodium options, establishing a supportive environment is a key opportunity to reduce sodium consumption. It is an important strategy, especially in higher-income countries where a major part of dietary salt intake comes from processed and restaurant foods [15,16]. For example, salt intake in hospital menus is frequently high; in a study in institutions across Canada, the mean sodium content in 86% of standard menus exceeded the safe upper level of 2300 mg [27,28]. When sodium is already present or used in food preparation, it cannot be removed after it is added. This therefore leaves the patient or consumer looking for lower-sodium options with less choice [29]. In the US, a toolkit for public health practitioners supports partnering with food service industries who supply public institutions to reduce salt in their food offerings. Effective strategies described include modifying preparation methods, using lower-sodium ingredients, product replacement, portion size reduction, and behavioural marketing [30]. This strategy is supported by 54 WHO member states, 41 of which have mandatory policies to reduce sodium intake for public food procurement and service [8].

### 2.3. Behavioural Change Communication

Ninety-six WHO member states have adopted mass media campaigns to sensitise populations about the risks of excessive salt intake [8]. Behavioural change communication and mass media campaigns have the potential to be highly complementary with other salt reduction initiatives and improve outcomes. Modelling of a strategy that combined industry agreements with public education to prompt people to consume 10% less salt over 10 years averted approximately 5.8 million DALYs/year related to cardiovascular diseases, at a population-weighted mean cost of I$1.13 per capita over the 10-year intervention, giving a cost-effectiveness ratio of approximately I$204/DALY. Furthermore, 96% of the global adult population lived in countries in which this intervention had a cost effectiveness ratio of <0.1 × GDP per capita [31].

### 2.4. Implementation of Front-of-Pack Labelling

Front-of-pack labelling is a key consumer awareness strategy that complements behavioural change communication and product reformulation. For example, food labelling is a requirement in the United Kingdom to inform consumers about the nutritional content of the food they buy [13] and must include the traffic-light scheme that displays red, amber, or green colour codes to indicate levels of fats, sugars, and salt [32]. In France, a similar, voluntary, scheme exists. Based on a composite for the nutritional value of that food, the Nutri-Score label gives a colour code from dark green with the letter A for “best nutritional quality” to dark orange with the letter E for “least good nutritional quality” [33]. Modelling analysis of the labelling scheme in the United Kingdom showed a gain of 1970 life years and a cost saving of £397,396,612 compared to no action over 10 years [13]. However, effects of communication and labelling initiatives may not reach the most deprived sectors of society [14], a factor which must be considered when designing policy interventions including these elements. To overcome this, simply-worded negative labels with warnings such as “high in salt” are easy to understand and are considered the most effective labelling system to date [34].

Front-of-pack labelling has been implemented by 40 WHO member states, though in only 12 of these countries is implementation fully or partly mandatory, mostly in the Region of the Americas [8]. Front-of-pack labelling may be particularly important in countries with a high consumption of ultra-processed foods (UPF). These foods often contain high amounts of sodium and have been associated with a higher incidence of hypertension and cardiovascular disease [35,36]. UPF consumption has been increasing in Latin America [36]. A mandatory front-of-pack labelling policy has been implemented in Chile using the simple wording “high in” for packaged foods that exceed 400 mg/100 g food. Along with simultaneous restrictions on food marketing to under-14-year-olds and restrictions on the school food environment, as well as an enforcement system with sanctions for non-compliance, this multi-component policy saw a decline in overall sodium content of purchases of 4.7%, and a decline in sodium purchased from “high in” products of 36.7% [37].

## 3. Iodine Fortification

Iodine is a trace element that is essential for the synthesis of thyroid hormone by the thyroid gland. Thyroid hormone is involved in growth, development, and the control of metabolic processes in the body. When iodine deficiency is severe, it can cause stunting, goitre and irreversible brain damage in the foetus and infant and retarded psychomotor development in children, though even when deficiency is less severe, general health and reproductive function is impaired, as well as children’s learning ability [36]. Collectively, these problems related to iodine deficiency are known as Iodine Deficiency Disorders (IDD).

### 3.1. Dietary Sources of Iodine

Much of the global soil is lacking in iodine and plant foods generally provide negligible iodine. Foods rich in native iodine are therefore limited in many populations. Rich sources include fish and seafood, algae, eggs, and milk from conventional dairy herds and other animals fed with iodine-fortified feed [37,38]. The use of iodised animal feed and iodophor disinfectants that augment iodine content in milk and eggs is permitted in conventional farming. However, these foods as reliable sources of iodine are hard to monitor and regulate due to seasonal changes in animal husbandry [39]. Furthermore, population-level changes in dietary preferences to either organic produce [restrictions on iodine use in feed and sterilization] [38], milk alternatives [40], or vegan diets [41] may also affect intakes from such sources. Drinking water typically has an iodine content of <10 μg/L though groundwater high in iodine has been revealed in regions of South America, Africa, China, and Europe, and may be the principal source of iodine for some populations [42].

### 3.2. Salt as a Fortification Vehicle

Since dietary sources of native iodine are limited, salt is accepted as an appropriate vehicle for iodine fortification, since (i) salt is widely consumed by virtually all population groups in all countries with little seasonal variation in consumption patterns, and intake is proportional to energy intake/requirements; (ii) in many countries, salt production is limited to a few centres, facilitating quality control; (iii) the technology needed for salt iodisation is well established, inexpensive, and relatively easy to transfer to countries around the world; (iv) the addition of iodate or iodide to salt does not affect the taste or smell of the salt or foods containing or cooked with iodised salt, and therefore consumer acceptability is high; (v) iodine [mainly from iodate] remains in processed foods that contain salt as a main ingredient, such as bouillon cubes, condiments, and powdered soups, and hence these products become sources of iodine; (vi) iodisation is inexpensive (the cost of salt iodisation per year is estimated at <0.06 US$ per person per year [43], and even less for established salt-iodisation programmes); and (vii) the concentration of iodine in salt can easily be adjusted to meet policies aimed at reducing the consumption of salt in order to prevent high blood pressure, heart attack, and stroke. Furthermore, though not currently recommended by the WHO [44], salt is used, or is under investigation for use for other fortificants. “Double-Fortified Salt” seeks to deliver iron alongside iodine [45] and salt is being considered for other single [46,47] and multiple micronutrients [48]. Salt has also proven to be a successful and cost-efficient route to deliver diethylcarbamazine, which is safe and effective at protecting individuals from the devastating effects of lymphatic filariasis [49,50,51].

### 3.3. Universal Salt Iodisation

Universal salt iodisation (USI) refers to the mandatory fortification of all food grade salt (sodium chloride) for human and animal consumption. This includes the iodisation of all salt for household use as well as salt used as an ingredient of processed foods and condiments [52]. USI is a mass fortification strategy that can meet population iodine requirements even in vulnerable groups with an increased need for iodine, such as pregnant and lactating women and infants [53]. In 1994, the Joint UNICEF/WHO Committee on Health Policy pressed for the global adoption of USI which, since its inception, has prevented an estimated 720 million cases of clinical IDDs, a 75% reduction from predicted rates [54]. Modelling suggests that USI prevents 20.5 million cases of iodine deficiency disorders in newborns annually and is highly cost-effective, leading to a global economic benefit of nearly $33 billion Net Present Value [54] for a cost of just 0.06 US$ per person per year, or less. Yet, despite the fact that many countries have adopted USI in response to the resolution passed in the 43rd World Health Assembly [55] that addressed the elimination of iodine deficiency disorders, it has been estimated that 1.88 billion people worldwide remain at risk of IDDs [56].

USI is the WHO-recommended strategy for the control of iodine deficiency disorders worldwide [52] and in 2019, 88% of people worldwide were consuming iodised salt [57]. Recommended iodine fortification levels are 20–40 mg iodine per kg salt, based on an estimated 5 to 10 g of salt intake per day in adult populations, and considered safe for individuals consuming up to 25 g salt per day when dietary iodine intakes are low [58,59]. Fortification levels should be adapted to population salt intakes and adjusted through routine monitoring. The WHO recommends an iodine intake of 150 μg per day for adults [59], which is achievable through the consumption of 5 g of salt iodised to 39 mg/kg or just 3 g salt if iodised to 60 mg/kg [52]. In some countries, there is voluntary or mandatory iodisation of other staple foods or condiments, including biscuits, milk, fish sauce, drinking water, yogurt, fruit beverages, seasoning powder, and bouillon cubes [60,61]. Population iodine supplementation using iodised oil is useful in emergency settings or where populations are severely iodine deficient and do not have access to iodised salt [62]. However, it is not considered a sustainable or cost-effective alternative to USI.

## 4. Salt Reduction and Iodine Fortification as a Common Public Health Agenda

The strategies to address excess dietary salt and a lack of native dietary iodine share a major thing in common: they are both of considerable public health concern as they both affect billions of people worldwide. Both are consequently major global public health priorities. Salt reduction and USI policies also share other commonalities in the following ways:

1. They are highly cost-effective interventions to improve health;
2. They have similar surveillance modalities;
3. They require complex negotiations with the food industries;
4. They depend on strong political support for optimum policy implementation;
5. They rely on the improved knowledge, attitudes, and behaviours of health care professionals;
6. They rely on increased public knowledge, education, attitudes and behaviours;
7. They rely on engagement of the salt manufacturing sector;
8. They are affected by a lack of food industry action;
9. They require a stable non-commercial funding source to be sustained.

It is clear from these points that salt reduction and salt iodisation should be synergistic [63]. Efforts by all stakeholders must be coordinated to create targeted interventions that cover the needs of all population groups, such that iodised salt is subject to the same salt reduction policy as non-iodised salt yet iodine intakes across all population groups are maintained.

These nine commonalities are expanded below.

### 4.1. Salt Reduction and Iodine Fortification Polices Are Highly Cost-Effective

Key policy interventions to reduce sodium intake include product reformulation, creating supporting environments for reduced salt consumption, behavioural change campaigns, and nutrition labelling initiatives. Together, these are considered cost-saving for reducing sodium intakes, costing ≤I$100 per DALY averted in LMICs [9]. At 0.06 US$ per person per year [43] and even less for established programmes, salt iodisation is a cheap intervention with considerable global economic benefits of nearly $33 billion [54]. The most cost-effective methodologies are population interventions rather than those targeting only affected groups (i.e., those with a diagnosis of hypertension or over a certain age, or iodine intake at-risk groups) [11]. Population-level approaches have inherent preventive benefits by reaching a wide amount of people with a comparatively low per-person cost.

If both salt reduction and USI, that is, the iodisation of all salt for human and animal consumption, are applied, these savings are potentially cumulative and can lead to cost savings [11].

### 4.2. Salt Reduction and Iodine Fortification Polices Have Similar Surveillance Modalities

Monitoring for salt reduction and salt iodisation are fully compatible and the coordination of monitoring and surveillance efforts for both salt reduction and iodine fortification policies is key to their success.

Both sodium and iodine status are easily assessed through dietary surveys and in urine samples that can be collected as part of routine health surveys, thereby necessitating minimal resource investment. Twenty-four-hour and casual spot urine collections can be used for sodium and iodine assessment, complemented by demographic and socioeconomic data, as well as comprehensive food surveys to distinguish the main sources of salt and iodine in the diet, particularly concerning the contribution of salt from processed foods and discretionary household use. Surveys should be repeated on a continuous cycle regularly, ideally every three to ten years [44,64].

Monitoring is essential to permit adjustments in salt iodisation over time, depending on observed salt intake in the population and in response to salt reduction policies, to ensure that individuals consuming the recommended amount of sodium continue to consume sufficient iodine [8]. Whilst a baseline survey before salt reduction polices are implemented should be prioritised wherever feasible, since these data will provide political power to enforce legislative approaches, salt reduction interventions should be implemented as a matter of urgency without a baseline sodium intake assessment as most populations are still consuming more than the maximum WHO-recommended intake of 2000 mg/day. With decreases in sodium intakes, iodine fortification can be easily adapted to fit the population, provided monitoring is in place. For example, the 20 mg/kg fortification limit applies, since many populations are consuming 10 g of salt per day. By reducing the salt consumption to 5 g, the iodine fortification would be adjusted up to 39 mg iodine per kg salt (Table 1 [52]).

#### Example of Coordinated Monitoring: South Africa

An example reporting the importance of coordinated sodium and iodine monitoring is provided by South Africa. South Africa mandated the iodisation of table salt in 1995, and revised fortification levels to 35–65 mg/kg in 2007 [65], a measure which has largely addressed iodine deficiency and IDDs in the country [66]. Later, in 2016, South Africa introduced legislation for maximum salt levels in common processed foods, being the first country to do so [66]. The legislation expected to decrease salt intakes by about 0.85 g per day, leading to predicted decreases in healthcare costs of US$51.25 million per year and out-of-pocket expenses for households of more than US$4 million per year. Yet, though data collected between 2015 and 2019 [66,67] suggest a decrease in salt intakes by 0.7 g, those with a salt intake of <5 g per day did not meet iodine intake requirements. In this case, further monitoring would be required to determine whether iodine fortification levels should be adjusted, especially since excessive iodine intakes have been previously documented in South Africa [68].

Routine monitoring also helps to avoid the over-iodisation of salt. The relationship between iodine intake and thyroid disorders is U-shaped [69] and excessive iodine intakes may lead to thyroid dysfunction [42]. Increases in incidence of hypothyroidism or thyroid autoimmunity in previously iodine-deficient areas may occur with the implementation of or abrupt changes in salt iodisation [70,71,72]; however, the effect is usually transient and can be minimised through robust monitoring and surveillance [42].

### 4.3. Salt Reduction and Iodine Fortification Polices Require Complex Negotiations with The Food Industries

The involvement of the food industry in salt reduction and iodine fortification can involve challenging negotiations. Market competition may lead to resistance to reformulated or new products [73], and thus, defining tangible targets and securing industry commitment is key [63]. Several large global companies such as Kellogg’s, Knorr, and Unilever have taken steps to significantly reduce salt in their food and beverage products, and their advertising of this is a significant leverage for smaller companies who do not have the same means, and has also led to institutional-level financial support [73].

Despite the challenges, product reformulation with either reduced salt or salt substitutes holds significant potential and according to various modelling scenarios provides the most return on investment across all interventions [13,14,74,75,76,77]. Reformulation is an ideal opportunity to introduce iodised salt where it was not previously used in a product and may be particularly applicable in LMICs as more processed foods are produced and consumed within the nutrition transition [17,78]. The addition of iodised salt to foods does not affect the taste or smell of the foods and therefore consumer acceptability is high. The private sector should be encouraged, with legislation [79,80], to harmonise salt content across staple foods, to eliminate competition and promote consumer taste adaptation [78] following WHO global sodium benchmarks [81]. It is critical that salt is not replaced by other unhealthful flavour enhancers such as glutamate, trans-fats, or sugar. Finally, the food industry should promote the benefits of eating reduced-salt foods through consumer awareness activities in food outlets, reduce salt in foods and meals served at restaurants and catering outlets, and provide clear labelling and signposting on the sodium content of foods and meals. Target setting for the amount of salt in foods and meals requires multisectoral actions with relevant ministries as well as support by civil society [9].

### 4.4. Salt Reduction and Iodine Fortification Polices Depend on Strong Political Support for Optimum Policy Implementation

A lack of political commitment has been identified as a core reason for the low priority set on food and nutrition interventions by national governments relative to a high disease burden [82,83]. Yet, government strategies should create environments that enable populations to consume adequate quantities of safe and nutritious foods that make up a healthy diet, including low salt [8].

Salt reduction and salt iodisation programmes are most likely to be successful if government-led [65] to ensure the engagement of all stakeholders. Many of the same stakeholders have been identified on national and international levels for both strategies, including international governmental and non-governmental organisations and interest groups, ministries of health and government public health and nutrition departments, funding organisations, health societies and associations, NCD coordinators, regulatory bodies, and the salt and food industries [46]. Bringing national and international stakeholders together could maximise resources and save costs, though this may be challenging when salt reduction and iodine fortification are spread across different departments and efforts to align initiatives may put strain on some relationships [46]. Efforts across all stakeholders must be coordinated if salt reduction and salt iodisation are to remain high on the public health agenda and not become lost within other public health campaigns [84].

The most successful strategies tend to involve some form of legislation. This needs to be designed carefully. Modelling suggests that food taxes can have unintended outcomes on purchasing preferences, prompting an increase in unhealthy substitute foods that are untaxed [85] or a decrease in fruit and vegetable intake [86]. In contrast, modelling studies that combined taxes for unhealthy foods with subsidies for healthier foods found that the benefits were increased [87]. Modelling suggests that taxing all foods based on their salt content is likely to have more impact than taxing specific products high in salt given that salt is pervasive in the food chain. Yet, achieving this impact is challenged by policymakers favouring taxation of specific products only [88].

### 4.5. Salt Reduction and Iodine Fortification Polices Rely on Improved Knowledge, Attitudes, and Behaviours of Health Care Professionals

Healthcare professionals (HCPs) are in a unique position to counsel and guide their patients and the public on healthy dietary actions. These include prompts to choose iodised salt, and advice on how to use less of it: “Use iodised salt, but less of it!”. For consumers, an understanding of recommendations, primary food sources, and the relationship between salt and sodium may be lacking [89,90]. HCPs can help to address this by discussing the benefits of sodium reduction on health and by providing practical guidance on how to lower sodium intakes in the home [90,91]. Consumers may also be confused by food labelling and HCPs can help with interpretation [89]. Barriers cited to providing such advice include prejudgements on a lack of compliance, other more important health issues for the patient, and a lack of time [92]. Nevertheless, advice on choosing and using iodised salt should be provided to all, regardless of blood pressure or other health issues or socioeconomic status, and where compliance issues are suspected, HCPs should adapt counselling to fit the patient, as receiving HCP advice is strongly associated with action [93].

Whilst the need for sodium reduction is generally clear for HCPs [92], some data suggest that there are gaps in the transfer of knowledge on iodine to the public by HCPs, especially during vulnerable life stages such as pregnancy [94]. Reports [95,96,97,98,99] of a low awareness and knowledge by HCPs on the importance of adequate intakes of iodine during pregnancy is of great concern and must be addressed.

### 4.6. Salt Reduction and Iodine Fortification Polices Rely on Increased Public Knowledge, Education, Attitudes, and Behaviours

In a survey of 192,441 adults in LMICs with high blood pressure, 26% had never had their blood pressure measured, 61% had not been diagnosed, and only 30% had received treatment, with 10% achieving control of their blood pressure [100]. This high prevalence of either undiagnosed or uncontrolled high blood pressure has significant implications not only for the health of the individual, but also carries substantial societal, developmental, and economic costs for LMICs [6]. Meanwhile, without awareness and information schemes, many people have little or no idea of the importance of iodine as a micronutrient and a poorer knowledge about iodine has been associated with a poorer iodine status [101,102]. Consumer awareness and the empowerment of populations through social marketing, education, and behaviour change campaigns are critical to raise awareness of the need to reduce salt intakes and also to sensitise people to the necessity of adequate iodine intakes [103], particularly during vulnerable life stages such as pregnancy and lactation and infancy [104]. Education schemes to empower the consumer to make healthier choices and take healthier actions can have powerful effects within salt reduction and salt iodisation policy. This is particularly applicable in LMIC settings where the primary driver for salt intake is discretionary use in the home [105]. However, education efforts must be sustained; even when public awareness campaigns are included with iodised salt initiatives, knowledge on the benefits of iodised salt may not remain complete [106].

### 4.7. Salt Reduction and Iodine Fortification Polices Rely on Engagement of the Salt Manufacturing Sector

Population monitoring for sodium status provides detailed information on how iodine policy must respond to changes in salt intakes. This requires engagement and flexibility from salt manufacturers. Whilst large-scale salt iodisation facilities may be easily able to reprogramme equipment and control and assure quality, this may be a challenge in LMICs. There, though iodised salt may be available in supermarkets, the small, local salt producer is rather the main supplier of household salt. Salt often purchased at the market, is often of low quality, and may not be iodised. Challenges for small producers include access to adequate iodisation equipment and the right kit and know-how to control the quality of iodisation. Solutions include purchase of such salt by larger organisations to sell for non-food use or for refining, processing, and correct iodisation [107].

### 4.8. Salt Reduction and Iodine Fortification Polices Are Affected by a Lack of Food Industry Action

Examples of where sodium reduction has been successful include the United Kingdom and Republic of Korea [34]. These policies have involved action across all stakeholder sectors, particularly the food industry, including sodium reduction or increased availability of low-sodium meals in restaurants, voluntary salt targets for product reformulation with timelines, strict monitoring, and a threat of legislation [34]. However, where there is a lack of engagement or interference from the industry, the success of policies is hindered.

Food industry action, as well as a strong political commitment and negotiation promotes the harmonisation of policies for foods for export. For example, for countries that share borders, a lack of cross-border harmonisation of salt iodisation policies may hinder the adoption of iodised salt in commonly consumed processed foods. In Switzerland, cheese is a major export. However, it cannot be exported to some countries when produced with iodised salt [40]. Policy makers should therefore be encouraged to harmonise agendas and engage the private sector to support the production of processed foods produced for export using iodised salt [80]. This includes packaging of foods for export; a regulatory capacity along with multisectoral action is needed to support labelling initiatives in salt reduction as well as iodine fortification [9,46,66].

### 4.9. Salt Reduction and Iodine Fortification Polices Require a Stable Non-Commercial Funding Source to Be Sustained

Whilst the engagement and buy-in of all stakeholders is key to the joint success of salt reduction and iodine fortification policies, a stable, non-commercial funding source will sustain results. The most successful policies are government-led [65], and though they involve the commitment of all stakeholders, when non-commercially funded, they are not dependent upon changing market forces or driven by marketing or consumer preferences. Funding originating from non-commercial entities such as international organisations, healthcare charities, or other independent associations provides the security and independence required to sustain strategies over the long term.

## 5. What Is Needed to Implement A Coordinated Salt Reduction–Iodine Fortification Policy?

Though there continues to be concerns about the implementation of both policies, with coordinated efforts and a harmonised approach, salt reduction is achievable whilst maintaining adequate iodine intakes across the population. So, what is needed to achieve such a coordinated salt reduction–iodine fortification policy?

The essential elements of successful programmes are political commitment, programme development with effective leadership and governance, effective partnerships with all stakeholders, and effective communication and advocacy [65]. Improving dietary habits is a societal as well as an individual responsibility. It demands a population-based, multisectoral, and culturally relevant approach [7,83].

### 5.1. Promoting Strong Political Commitment

Political commitment can be created and built upon over time through strategic actions. Members of the Ministries of Health need to get behind and champion coordinated salt reduction–iodine fortification. Lobbying from health associations and other civil society movements as well as the media promotes political commitment to support the common goal of a coordinated salt reduction–iodine fortification policy [82]. This coordinated policy spans not only nutrition and health, but trade, education, and relationships with the private sector. These policies are complementary and synergistic, and political backing should use and adapt existing structures and systems for maximum effect [108].

### 5.2. Strong Programme Leadership and Governance and Effective Policy Development

Effective policy development is key to securing operational commitment and ensuring that rhetorical commitment from government and institutional commitments from policymakers turn into tangible actions. Here, alongside the development of policies, strong monitoring and regulatory aspects must be integrated to ensure sustained success. This could be best achieved by the implementation of individual[s] recognised as leaders within nutrition and situating them in prominent positions inside or outside of government, to guarantee ongoing commitment from those both above and below in hierarchical organisations [109]. Such individuals can lobby for and influence the allocation of adequate financial resources to be directed towards coordinated salt reduction–iodine fortification policies, as well as manage external actors that fund health policies and influence implementation, particularly in LMICs [109]. Such external actors, possibly international organisations or similar, may help to provide technical assistance and legitimacy to policy initiatives and support in government advocacy. However, their roles must be aligned with government priorities for maximum effect [83].

Precise, culturally applicable salt reduction–iodine fortification policies should be informed by up-to-date and relevant evidence, and governments must be equipped with the right knowledge and capacity to support action [108]. Scenario modelling can help to inform policy development, as well as pilot studies to assess the nutrition landscape.

Salt reduction–iodine fortification strategy is reliant on robust monitoring and surveillance planning included in the policy from the outset. At initiation, plans should be in place for routine evaluation every three years [46,66]. Monitoring should include dietary assessment through questionnaires, as well as urinary sodium and iodine analysis, and may include the engagement of academia to help prioritise research on optimal dietary targets and cost-effectiveness analysis of policies [108].

### 5.3. Effective Partnerships with Stakeholders

Partnerships improve the possibility of achieving coordinated salt reduction–iodine fortification policies. The private sector is a major stakeholder that requires engagement for action on the use of iodised salt in processed food products and salt reduction and/or substitution efforts as well as front-of-pack labelling. Voluntary and mandatory regulations have been most successful in demonstrating cost-effectiveness where intervention is across several levels e.g., labelling alongside product reformulation.

Where leading world food producers in the private sector have reduced salt, advertising is strong, and strengthened by the inclusion of messages on the importance of iodine where the foods are made with iodised salt. This may promote consumer awareness and purchasing demand, as well as prompting smaller food manufacturers to follow suit. Private sector stakeholders should be prevented from establishing outlets close to schools, hospitals, workplaces, or other public institutions, unless they provide a supportive environment for the consumption of low-salt alternatives and use iodised salt within a healthy or health-promoting product offering.

However, conflicts of interest can arise between the different stakeholders. This should be managed by strong program leadership and clear rules of engagement [108].

### 5.4. Effective Communication and Advocacy

Such educative initiatives are cost-effective initiatives for both salt reduction and iodine fortification policies. When brough together in a coordinated policy, the message is very simple: use iodised salt, but less. This concept can be promoted on all levels. Policies can be designed with simple but effective slogans. HCPs can be provided with training and ongoing updates covering the benefits of reduced sodium and adequate iodine intakes, and how to translate this into practical guidance for their patients, e.g., the use of spices in cooking or salt alternatives to add at the table. Food purchasing is positively influenced by front-of-pack labelling [89], especially when labels are simple and easy to read, understand, and interpret, such as “low in salt and rich source of iodine” [34]. Furthermore, front-of-pack labelling campaigns can be strengthened through complementary educative messages transmitted in parallel through social media. mHealth can capture data on purchasing preferences and salt consumption whilst transmitting health-promoting ideas [110].

Communication and advocacy should involve all relevant stakeholders, from public interest groups and health associations; the private sector, including manufacturers, distributors, and vendors; food brokers and salespeople; group purchasing organisations; those producing and serving foods such as contract food services, chefs, and service staff; through to HCPs, including doctors, nurses, midwives, pharmacists, dieticians, and nutritionists, as well as those working in nutrition surveys.

Education initiatives should include revisions of dietary guidelines, where necessary, to bring both salt reduction and adequate iodine intakes to the forefront and accompanied with relevant messages [use iodised salt, but less]. Education should also start with school curriculums that include courses on nutrition and culinary skills that explore the use of techniques to lower the use of salt in cooking and improve dietary diversity. Education in the home, e.g., through midwives and healthcare visitors, can help to improve nutritional choices in pregnancy, early infancy, and childhood, thereby providing the child with a good start with adequate iodine and a taste not accustomed to high salt intakes.

## 6. Conclusions

Excessive salt consumption is a major contributor for high blood pressure and increases the risk of heart attack and stroke. Many countries still have average intakes above the WHO target of <5 g salt per day. With the nutrition transition and double burden of malnutrition increasing around the world, high blood pressure, heart attack, and stroke are now also an urgent issue in low- and middle-income countries. Global progress in the adoption of salt reduction policies by WHO member states has been recently summarised by the World Health Organisation [8], who conclude that salt intakes could be dramatically reduced by 2030. Such a reduction would, however, depend upon accelerated policy adoption to include at least two mandatory salt reduction interventions, as well as implementation of best practices [8], so there is clearly work to do to reach this modelled scenario.

Iodine deficiency remains a risk for several countries worldwide and predictions state that 4.8 million newborn infants will be affected by iodine deficiency disorders [54]. Despite the risks from excessive salt intake, salt remains an ideal vehicle for population iodine fortification, particularly as it can be included ubiquitously in staple foods, including bread and bouillon, across the food chain. Therefore, public health policy must find a way to integrate these policies successfully.

The recommendations outlined in this review should be considered alongside country-level data on sodium and iodine intakes, the inclusion of which is beyond the scope of this manuscript. In conclusion, through a coordinated approach and joint planning, salt reduction–iodine fortification policies are an effective, cost-efficient, and achievable double duty opportunity to influence health outcomes for present and future generations. Countries are urged to act without delay to bring salt reduction and iodine fortification together.

## Figures and Tables

**Table 1:** Suggested concentrations for the fortification of food-grade salt with iodine [52].

Estimated Salt Consumption g/Day ^1^	Average Amount of Iodine to Add, mg/kg Salt (RNI + Losses) ^2^
3	65
4	49
5	39
6	33
7	28
8	24
9	22
10	20
11	18
12	16
13	15
14	14

^1^ This includes consumption as table salt as well as salt from processed foods. ^2^ This fortification concentration was calculated based on the mean recommended nutrient intake of 150 μg iodine per day + 30% losses from production to household level before consumption, and a 92% iodine bioavailability. Losses depend on the iodisation process, the quality of table salt, the packaging materials, and the climatic conditions. Losses could vary widely, and this table presents the value considering 30% losses. The monitoring of urinary iodine concentrations will allow for adjustment of the selected fortification concentrations. RNI: recommended nutrient intake. Shaded areas correspond to the World Health Organisation sodium intake reduction guideline.
